# An investigation of the impact of ‘Living with COVID’ on workplace COVID-19 transmission risk, response and resilience - lessons learnt and future challenges

**DOI:** 10.1186/s12889-024-20320-3

**Published:** 2024-10-18

**Authors:** Catherine Lewis, Sheena Johnson, Claire Mann, Janet Ubido, Bernadine Farrell, Anna Coleman, Martie van Tongeren

**Affiliations:** 1 Division of Population Health, Health Services Research & Primary Care, University of Manchester, Manchester, UK; 2 Alliance Manchester Business School, University of Manchester, Manchester, UK

**Keywords:** COVID-19, Employment, Employees, Employers, Local authorities, Mitigating strategies, Resilience

## Abstract

**Background:**

Previous research has highlighted links between occupation and risk of COVID-19 transmission and suggests that occupational risk is influenced by covariates including socio-economic status, and deprivation. This study examined the perspectives of local authority teams of how changes in policy and advice, as set out in the UK government report ‘Living with COVID,’ affected COVID-19 transmission risk, response and resilience in workplace settings in Greater Manchester, an area that was disproportionately affected by the pandemic.

**Methods:**

The project, which took place between August and November 2022, undertook a mixed methods approach to incorporate wide-ranging reflections of changes following the publication of ‘Living with COVID’. Quantitative data was collected from local employers (*n* = 149) and employees (*n* = 397) using online surveys, and qualitative interview data was collected from Greater Manchester local authority teams (*n* = 19).

**Results:**

The research highlighted the inequitable impact of the pandemic on those already experiencing health inequalities, including people on more precarious employment contracts or those who were unable to work from home during the pandemic.

The study found that the facilitators that helped local authorities to support employers to manage transmission included clear, detailed and timely national and local guidance, good communication, partnership working, funding, and timely access to data. Barriers to supporting employers included contradictory or confusing national guidance, structural inequalities, lack of funding, and delayed access to data. Interview participants reported that they were now utilising lessons learnt during the pandemic, along with the that partnerships developed, in order to tackle wider health issues and to prepare for future pandemics or health crises.

**Conclusion:**

The findings of the study, which concur with previously published research conducted as part of the PROTECT project, highlight the importance of active reflection on the lessons learned during the course of the pandemic. The study draws on PH and EH teams’ perspectives of managing COVID-19 transmission, in an area that was disproportionately affected by the pandemic, in order to add to our understanding of the best ways to ensure preparedness for future pandemics or health crises at a national level.

**Supplementary Information:**

The online version contains supplementary material available at 10.1186/s12889-024-20320-3.

## Background

Previous research, including work that was conducted as part of the PROTECT National Core Study on Transmission and Environment (PROTECT) as well as wider literature, has highlighted links between occupation and risk of COVID-19 transmission [1–4]. Research conducted by Beale et al. [1] within the framework of PROTECT, for example, suggested that occupation influences COVID-19 transmission risk in a number of ways. Occupation impacts workers capacity to engage in remote work, maintain social distancing in the workplace, operate in adequately ventilated settings, and obtain suitable personal protective equipment (PPE). Analysis conducted as part of the PROTECT project also suggested that people working in certain sectors, including health, social care and transport were at higher risk of infection during the pandemic. Research conducted as part of PROTECT as well as wider literature also suggests that the ability of individuals to isolate, when necessary, along with workplace policies such as sickness absence and provision of sick pay influences workplace transmission of COVID-19 [3, 4].

There is evidence indicating that occupational risk is influenced by covariates including ethnicity, socio-economic status, and levels of deprivation [4–6]. This study builds on a previous study by the authors of this study which included interviews with Directors of Public Health (DsPH) [4]. The previous study examined DsPH’s opinions on why certain local authority areas had experienced high, sustained levels of COVID-19 prevalence, and been designated by the Scientific Academic Group for Emergencies (SAGE) as ‘areas of enduring prevalence’ [5]. DsPH suggested that 'enduring prevalence’ of COVID-19 was likely to be caused by the interaction of several risk factors, including deprivation, factors related to employment including inability to self-isolate, and living in overcrowded housing. DsPH also highlighted the influence of the convergence of these structural factors with demographic factors such as age and ethnicity on wider transmission rates. Wider research, such as a study conducted by Daras et al. [6] also identified clustering of risk factors for COVID-19 transmission within specific geographical areas. For instance, workers who do not receive sick pay or are on low incomes may also reside in more deprived areas, increasing their COVID-19 transmission risk.

The literature suggests that the COVID-19 pandemic had a more pronounced impact on the population, economy, and health of Greater Manchester (GM) than England as a whole. For example, two of the ten ‘areas of enduring COVID-19 prevalence’ identified by SAGE [5] were in GM. The 2021 Census [7] revealed that GM’s demographic makeup differed from that of England overall in several aspects, such as age distribution, ethnic diversity, education and employment patterns.

### Demography of greater manchester

According to the 2021 Census [7] around 2,867,800 people lived in the area covered by GM Combined Authority in 2021. According to the Census the percentage of females in GM (50.7%) was slightly lower than England as a whole (51.0%) whilst the percentage of males was slightly higher (49.3% and 49.0% respectively). In terms of ethnicity, the percentage of people who described their ethnicity as ‘White’ was lower in GM (76.4%) than in England as a whole (81.0%). The percentage of people who described their ethnicity as ‘Black, Black British, Black Welsh, Caribbean or African’ was higher in GM (4.7%) than in England as whole (4.2%), along with the percentage who described their ethnicity as ‘Asian, Asian British or Asian Welsh’ (13.6% and 9.6% respectively).

Overall, the population of GM was younger than that of England as a whole, according to the Census. For example, there was a slightly higher percentage of people aged 20–24 (6.6% compared to 6%), aged 25–29 (7.1% compared to 6.6%) and aged 30–34 (7.4% compared to 7.0%). There was a slightly lower percentage of people aged 50–54 (6.6% compared to 6.9%), 55–59 (6.3% compared to 6.7%) and 60–64 (5.3% compared to 5.8%) in GM than in England as a whole.

Household deprivation was greater in GM than nationally. The percentage of households who were ‘not deprived in any dimension’ was 45.8% in GM, lower than the national average of 48.4%. The percentage of people who were in good or very good health in GM (81.2%) was slightly lower than in England as a whole (82.2%), whilst the percentage of individuals who were in bad or very bad health in GM (6.0%) was slightly higher than in England as a whole (5.2%). According to 2021 statistics the percentage of the population aged 16 and over who had no qualifications in GM (20.0%) was higher than England as a whole (18.1%). The percentage of individuals in GM with level 4 qualifications (such as NVQ level 4 or higher apprenticeships) and above (for example graduate diplomas or degree apprenticeships) was 31.9%, which was lower than the national average of 33.9%.

In terms of employment, the percentage of individuals who reported that they worked in ‘higher managerial, administrative and professional occupations’ (11.3% compared to 13.2%) and ‘lower managerial, administrative and professional occupations’ (18.7% compared to 19.9%) was lower in GM than in England as a whole. The percentage of individuals who reported that they worked in ‘routine occupations’ (13.1% compared to 12.0% in England as a whole), had ‘never worked’ or were long-term unemployed (10.9% compared to 8.5%) or were full-time students (8.8% compared to 7.7%) were higher in GM than in England as a whole. Regarding employment sectors, a higher percentage of people in GM than in England worked in ‘sales and customer service occupations’ (8.7% compared to 7.5%) and ‘process, plant, and machine operatives’ (7.3% compared to 6.9%).

In terms of economic activity, the proportion of the population who were economically inactive was slightly higher in GM (40%) than in England as a whole (39.1%). The proportion who were in employment was lower in GM (56.1%) than in England as a whole (57.4%) and the proportion who were unemployed in GM (4%) was slightly higher than in England overall (3.5%).

### Exacerbation of existing inequalities in health

As seen in previous disasters, across England groups who were the most severely affected by the COVID-19 pandemic were those who were already the most disadvantaged [8]. Researchers have emphasized the importance of recognizing how factors including ethnicity, disability and class are closely intertwined and influence one another within society [9]. Groups that were disproportionately affected by the COVID-19 pandemic included certain occupational groups, as well as people living in areas of high deprivation, older people, people with disabilities and minority ethnic groups [10, 11]. Although these socio-economic inequalities in impacts of the COVID-19 pandemic were seen nationally, the differences were more pronounced in GM than in England as a whole [12]. Marmot et al., [13] suggested that health inequalities in GM had already begun to increase prior to the pandemic, but that the pandemic exacerbated these existing inequalities in health [12].

### Local authority support for GM workplaces during the course of the COVID-19 pandemic

Throughout the pandemic, there was pressure on workplaces to ensure that the risk of COVID-19 infection was reduced. In response to this, local authority public health (PH) and environmental health (EH) teams provided support for GM employers throughout the pandemic, in order to help them control workplace transmission. There have been a number of changes to government policy on the management of COVID-19 throughout the course of the pandemic, including the publication of the government report ‘Living with COVID-19’ [14] in February 2022. ‘Living with COVID’ [14] suggested that the future path of the virus was currently uncertain. It therefore emphasised the importance of future planning in order to maintain resilience in order to be prepared for possible resurgences of the virus or the emergence of potential future variants.

Building on previous work that was carried out as part of the PROTECT project, this study explored the perspectives of local authority PH and EH teams of how changes in policy and advice, as set out in the UK government report ‘Living with COVID,’ affected COVID-19 transmission risk, response and resilience in workplace settings in GM.

## Methods

The current study, which was conducted within the framework of PROTECT, used a mixed methods approach to examine the impact of ‘Living with COVID’ across the GM area [15]. A convergent parallel design was used for the study [16]. Quantitative data was collected from local employers and employees using self-administered online surveys which were conducted via Qualtrics and analysed using SPSS. Qualitative data was collected from stakeholders, primarily PH and EH teams, who were working in local authority roles in the region.

Two online surveys were carried out between August and October 2022. The first was focussed on employers and senior managers and the second was focussed on employees. Inclusion criterion for all participants was that they worked in GM workplaces during the pandemic. Additional Files 1 and 2 contain the lists of questions that were included in the employer and employee surveys.

Qualitative data was collected from stakeholders working in local authority roles in GM. The whole study population was people working in local authority PH and EH roles in the ten GM local authorities. A total of 19 participants took part in 14 semi-structured interviews and three focus groups consisting of between two and three participants. Focus groups were used when participants asked if they could take part in an interview with their colleagues from the same local authority area, in order to generate discussion between the participants [17]. Inclusion criteria was participants who held senior roles in managing the COVID-19 response in local authorities in GM. Purposive sampling, where participants who were able to provide the most relevant information in order to meet the research objectives [18] was used along with snowball sampling [19] in order to ensure that all ten boroughs in the region were represented. Initially, DsPH for all GM local authorities were contacted via email to ask if they would like to take part in the research. DsPH were asked to suggest other key PH and EH contacts within their local authority areas. EH participants then asked their counterparts in other GM local authorities to contact the researchers if they would like to take part in the research. In order to safeguard anonymity each respondent was given a unique ID code. A full list of codes is provided in Additional File 3, along with a list of participant roles. Participants were given an information sheet detailing the project and were asked to sign a consent form to confirm they had provided informed consent before participating in the research [20] The information sheet and consent form are provided in Additional Files 5 and 6.

The interviews were conducted online via Teams or Zoom by two researchers between August and November 2022. They included 15 questions and lasted for an average of one hour. Respondents were asked to identify the main factors they believed contributed to differences in workplace outbreaks and transmission of COVID-19. They were asked for their opinions of the effectiveness of local, regional and national level strategies, guidance and policies in preventing workplace COVID-19 transmission during the pandemic, along with barriers and facilitators to preventing transmission. The full list of interview questions, along with prompts used, is provided in Additional File 4.

All interviews began by explaining the purpose of the research and asking if participants had any questions about the project. All interviews began by asking participants about their role, however as semi-structured interviews were being used, the order of the questions could be varied. Although prompts were used, additional questions could also be added if needed [21].

Interviews were conducted until data saturation was reached and new themes were no longer being generated by the interviews [22]. Following the interviews, the qualitative data underwent verbatim transcription. Data was analysed using thematic analysis [23] by two researchers experienced in using qualitative methods, using NVivo analytics software. This was an iterative process – during the initial coding phase, the codes were continually revised based on discussions between the researchers. A third of the interviews were also second-reviewed by the wider research team to reduce the potential for bias during the coding process and to meet standards of rigor in conducting qualitative research [24].

Both deductive and inductive analysis methods were used to analyse the data [25]. The initial coding framework was shaped by the research questions and issues raised by participants during the interviews. Deductive methods were used in order to identify key aspects of the data in order to answer the research question. Inductive methods were employed to uncover emerging patterns in the data set, providing insights relevant to the research topic.

The study received ethical approval from University of Manchester Proportionate Research Ethics Committee (Ref: 2022–14470-24900).

The next section of the report presents the findings derived from the online surveys and interviews conducted with key stakeholders, followed by an overall discussion and recommendations.

## Results

This section of the report provides an overview of the findings that were derived from empirical data collection. The first section summarises the findings of the quantitative online surveys for employers and employees. Following that, the second section reviews the qualitative data collected from key stakeholders holding roles within local authorities.

### Quantitative data—GM area employee/employer survey

The employer survey received 96 valid responses. Employers from a diverse range of sectors participated in the survey, with manufacturing comprising the largest proportion at 15.6%, followed by 'other service activities’ at 12.5%. The employee survey received 397 valid responses. Around four in five respondents to the employee survey (79.4%) held a qualification at degree level or above, and almost 71.3% of respondents to the employee survey worked in the education sector.

**Fig. 1:**
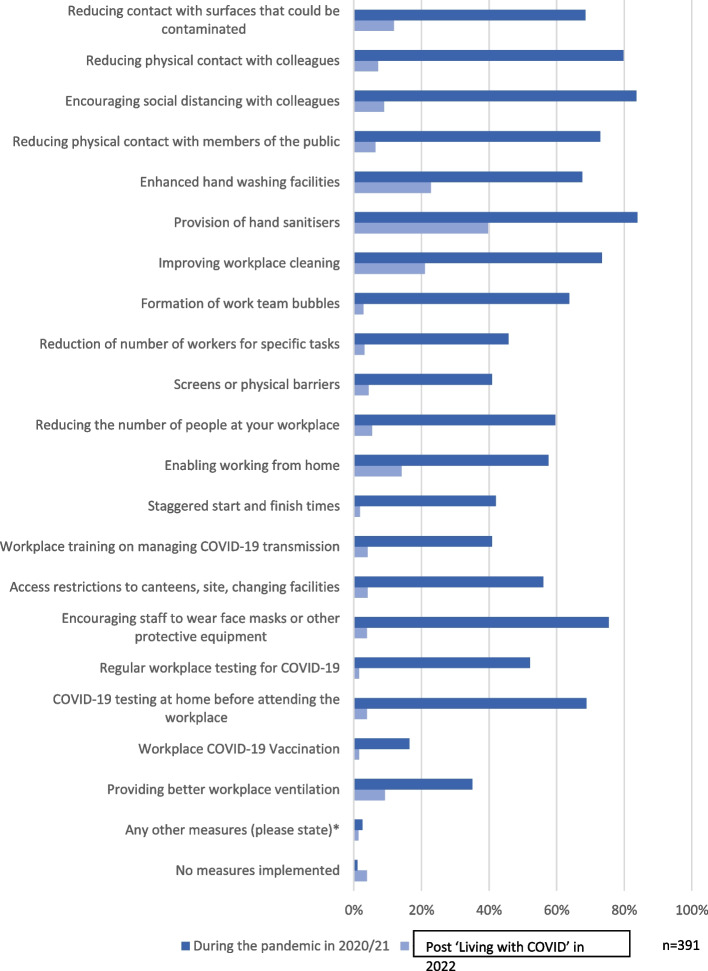
Which measures did your employer implement to reduce the risk of infection?

The employer and employee surveys indicated that mitigation measures implemented during the pandemic had been significantly reduced by the time the surveys were conducted. For example, 70% of those completing the employee survey reported that 'Encouraging social distancing with colleagues', 'Reducing physical contact with colleagues' and 'Encouraging staff to wear face masks or other protective equipment' had all been discontinued. However, over a third of respondents reported that their employers continued to provide hand sanitisers (39.6%). Other measures that were more likely to remain included enhanced handwashing facilities (22.8%), improved workplace cleaning (21.0%) and enabling working from home (14.1%) (Fig. 1).

Amongst employers, just under half (47.9%) reported that wearing of face masks at work was encouraged during the pandemic, reducing to 13.5% at the time of data collection. Three quarters of employees (75.5%) reported that wearing of face masks at work was encouraged during the height of the pandemic in 2021, which was reduced to less than 5% post ‘Living with COVID’ in 2022.

The majority of employees strongly agreed they could take time off to self-isolate if needed. However, 5.9% of employees reported attending work during the pandemic despite being asked to self-isolate. For those earning less than £15,000, this figure rose to 9.1%—higher than all other income groups except for the £50,000 to £59,000 bracket, where the figure was 21.1%.

The study aimed to explore if vaccination rates were lower in certain sector or among certain groups. However, the employee survey showed that vaccination rates overall were high – only 3.8% of respondents (*n* = 15 of the 391 respondents who answered the question) reported that they were unvaccinated. The employee survey also revealed that reported levels of self-diagnosed Long COVID were high, with almost 1 in 4 (23%) describing themselves as having the condition.

The study also aimed to explore the impact of the COVID-19 pandemic on workplace stress. More than two thirds (68.8%) of employees found their job extremely or very stressful during the pandemic, compared to less than a third (29.8%) prior to the pandemic. At the time of data collection levels of stress had decreased but remained elevated compared to pre-pandemic levels, with 46.4% of employees finding their job very or extremely stressful.

### Qualitative data - local authority stakeholder views

This section encompasses participants’ opinion of areas of increased risk, in terms of their role in supporting employers to manage COVID-19 transmission and outbreaks during the pandemic. PH participants included DsPH, PH Specialist Leads and PH Consultants. EH participants included Regulatory Services Managers and Environmental Health Officers (EHOs) (see Additional file 3).

This section includes an overview of the local authority COVID-19 response, outlining how local authority PH and EH teams supported employers during the pandemic. It also provides an overview of the facilitators and barriers encountered by participants whilst supporting employers. Participants’ opinions on the post ‘Living with COVID’ situation are presented, along with their reflections on the lessons learnt during the pandemic and perceived future challenges. Anonymous quotations from participants are used for illustrative purposes.

### Risk factors identified by interview participants

Participants identified a number of factors that they felt were related to increased risk of workplace transmission of COVID-19. These included socio-demographic factors –participants highlighted that GM experiences higher levels of deprivation than the national average. Participants also drew attention to the increased risk of transmission related to workplace risk factors including provision of sick pay and ability to work from home. Schools and care homes were also identified as areas where there was a heightened risk of COVID-19 outbreaks, especially by PH participants who had these organisations within their remit.

### Socio-demographic factors

Numerous participants emphasised the connections between socio-economic factors and transmission of COVID-19. They highlighted that workers who would not get paid if they did not work, including those who were unable to work from home, were less likely to isolate if needed. This included employees who did not receive sick pay, or were self-employed but did not receive support grants. It also included workers who were on more precarious employment contracts including agency workers and workers on zero hours contracts. Workers who would not get paid if they tested positive for COVID-19 and subsequently stayed away from work, especially frontline workers, might also hesitate to take a test, thereby heightening the risk of COVID-19 transmission in the workplace:

*“Potentially what we were seeing is a difference in propensity to test between different populations with those people who had a lower… personal consequences if they tested positive being more likely to test and test regularly and report those results compared to populations who perhaps were doing more frontline work or face to face work and had more consequences if they couldn’t go out to work.” (*P1, PH*)*

Participants also asserted that workers who were in insecure or low paid work were also more likely to reside in the more deprived geographical areas or wards within the local authority, further increasing their vulnerability to COVID-19 transmission. Many participants highlighted certain geographical areas or wards within their local authorities where there had been persistent, high rates of COVID-19 transmission. Participants emphasised factors related to employment as a reason for these disparities in the risk of COVID-19 transmission, alongside other risk factors such as residents living in overcrowded housing:

*“And you saw that with the inequalities in the uptake of testing when Liverpool piloted mass testing, and saw that uptake was much higher among the more affluent parts of the city. So I think that is probably the single biggest factor as it relates to workplaces.” (P14, PH)*

Participants also reported that certain groups (e.g., people from certain age groups, such as young people) might be at greater risk of transmission. This vulnerability stemmed from their greater likelihood of members of these groups occupying frontline roles where remote work was not feasible and where sick pay might not be provided. They also reported that vaccination rates were lower among certain groups of people, which increased their risk of worse health outcomes if they contracted COVID.:

*“I remember us looking at taxi drivers and security guards, they were coming up quite often as well, and we’d often find that they were from our ethnic minority groups that again were at highest risk, they weren’t vaccinated, they were in these... front-facing jobs and were in a lot of contact with people and they were vulnerable.” (*P7, PH*)*

Numerous participants also discussed high prevalence of residents with long-term health conditions, as well as those leading unhealthy lifestyles, within their local authority areas. This also increased workers’ risk of suffering worse health outcomes if they were to contract COVID-19:

*“For a number of reasons…we experience quite poor health outcomes, probably more so than you would expect given our overall level of deprivation. Certain areas in terms of things like alcohol, cardiovascular disease… cancers, particularly lung cancer and other respiratory disease.”* (P10, PH)

Whilst funding acted as a facilitator to enable local authority teams to support workplaces, funding was often not sufficient to address the risk factors outlined above, which included lack of sick pay and inability to work from home.

### Schools and care homes

Multiple participants highlighted that schools and care homes required more focus than other workplaces. The reasons suggested by participants for this need for this increased focus included the relatively high number of COVID-19 outbreaks in these workplaces. However, participants noted that this was somewhat moderated by the higher levels of testing that was conducted in these settings compared to other workplaces:

*“I think one of the things that we saw around schools, certainly through the first couple of waves, so definitely through 2020 and possibly through the first part of 2021, was the bulk of transmission. Because with schools and care homes you had higher rates of testing than…so we were able to get a better sense of what was going on in those settings than perhaps we would have in some other workplaces.*” (P1, PH)

Multiple participants also emphasised the increased risk of serious disease, due to age and co-morbidities, to residents of care homes if they contracted COVID-19, compared to younger adults. They highlighted that the support required in these settings from PH and EH teams was generally resource intensive for the teams. In the case of schools, for example, this involved liaising with a large number of individual head teachers. Participants identified a number of structural barriers to controlling COVID-19 transmission in these settings. For example, participants highlighted the care sector as one where people were less likely to receive sick pay. As discussed in the previous section on socio-demographic factors, this might then impact workers’ ability to isolate when needed. Additionally, participants reported that workers in the care sector experienced relatively high levels of deprivation compared to those in other professions:

*“Some of our care home staff…we know that a large chunk of those people don't get paid if they don't go in. And we know that there’s work … that's come out recently that shows that quite a high proportion, compared to other professions and other jobs, of people working in the care sector are living in relatively higher levels of deprivation.” (*P13, PH*)*

### Local authority response

#### Workplace interventions

Participants discussed implementing a wide range of measures in order to support employers to manage COVID-19 transmission and outbreaks during the course of the pandemic. This included support with contact tracing, testing, and vaccination. As shown in the quote below, for example, participants discussed setting up vaccination clinics outside larger workplaces, in partnership with NHS teams:

*“We actually then brokered surge vaccination in some particular car parks that were adjacent say to our food production factories. …we developed real collaborative working with our NHS vaccination team, not only did we have surge testing, but we had surge vaccination... And so, we were actually able to serve that workforce community and also provide the advice and guidance.” (P9, PH)*

Additionally, participants discussed implementing more targeted interventions for groups of workers who were seen as higher risk, including the implementation of COVID-19 risk health checks:

*“So, we also put in COVID-19 risk health checks, so we were able to give that offer when there wasn’t vaccination yet. And those COVID-19 risk health checks are still running…they were for people with a long-term condition, or from a BAME (black or minority ethnic) community. Taxi drivers, care workers, et cetera, I mean, when you look at the differential impact on the most disadvantaged in our communities, they are the ones who continued to work in frontline services right through lockdown.”* (P11, PH)

Several participants emphasised the critical role of workplace ventilation in mitigating transmission risk, and emphasised the importance of supporting employers to improve ventilation:

*“Obviously, ventilation was one of the main things that kind of came in a bit late in the day, but it was that focus more in 2021 that under health and safety legislation we were trying to make sure that…workplaces were well ventilated, which they should be anyway, but even more so to control the spread.” (P3,* EH)

Several participants reported supporting employers in addressing the mental health needs of their workforce during the course of the pandemic. The move to remote working presented challenges, such as isolation, for some workers:

*“We were able to put into place quite a robust package of support for staff…Then combining that as well with the wellbeing support…Around them obviously working from home and that feeling of being isolated, ensuring they still felt part of an organisation, they still had that contact with the managers and the teams. So, there was a whole raft of work around that that was a quite resource-intensive but important aspect certainly at the beginning of the pandemic, but then continued through it as well as people continued to work from home for longer periods of time.”* (P5, EH)

### Facilitators and barriers to supporting employers to manage transmission

Participants identified a range of strategies, including national guidance, good communication, timely access to data and sufficient funding, which helped them to support employers to manage workplace transmission and outbreaks of COVID-19. However, participants also identified issues relating to these measures (e.g. confusing or contradictory national messaging, poor communication, delayed access to data and insufficient funding) as barriers to supporting employers in managing transmission and outbreaks.

### Facilitators

#### Helping employers interpret national guidance

Many participants reported that a substantial aspect of their role involved helping employers to comprehend government guidance and to put measures into practice. Respondents who found national guidance useful reported that it was produced at a fast pace, and that it was more prescriptive than the legislation that they normally encountered, thus facilitating enforcement measures:

*“In many ways, the regulation was more prescriptive than much of the legislation we would normally deal with and then was backed up with the guidance that was very prescriptive as well. And that was really helpful for us when speaking to businesses.” (*P4, EH)

As well as providing advice on preventative measures, PH teams also provided businesses with support with contact tracing and COVID-19 testing, in order to control outbreaks and to reduce the risk of future outbreaks.

#### Communication

Overall, good quality messaging and communication strategies, cascaded not only from government to local authority but also from local authority via the PH function to local businesses, were seen as facilitators of reducing COVID-19 transmission:

*“I did a lot of engagement with business leader groups…I’d go along to give them some advice and reassure them or give them an update…or actually paint the picture of doom which was necessary in some cases … trying to put it in the terms of if you end up with more staff off sick or if you end up with a big outbreak, and we have to take further steps it's not good for your business either. So, there was quite a bit of that preventative work we tried to do and getting out there with comms and promotion of the testing offer or the vaccination offer.”* (P10, PH)

#### Partnerships

Many participants emphasised the advantages of partnership working at local, regional and national levels as a facilitator of managing the risk of COVID-19 transmission. Participants highlighted close collaboration with a wide range of stakeholders including local police, clinical commissioning groups (CCGs), and with local communities, including with community and voluntary organisations and with faith leaders. Additionally, participants discussed partnership working within communities to reduce transmission as well as between employers (such as different call centres) working together to discuss strategies to reduce transmission.

Many participants discussed close partnership working between PH and EH teams, which, for some participants, entailed daily online meetings during the peak of the pandemic. PH participants also discussed consulting with EH colleagues when they needed advice on specific issues such as ventilation:

*“For example, environmental health have been involved in some of our nursery outbreaks, because they could look at the scheme of...the ventilation requirements et cetera, in a way that, of course, we weren’t trained to do.”* (P12, PH)

Some participants described partnership initiatives that were focussed on specific issues, such as PPE, whilst others reported concentrated efforts on interventions in settings that were high risk, such as schools and care homes:

*“I also had to work to establish what we called a COVID-19 hub which also had nurses in but also patient advisors, we set up a helpline number, we had schools reporting directly into us, so we set up the systems around all that so that our infection control nurses could focus on care homes and other high-risk settings like our asylum seeker hotel and things like that.” (*P7, PH)

As discussed later in the ‘reilience’ section, participants highlighted that many of the partnerships that had been developed during the course of the pandemic had been sustained and broadened in order to focus on wider health issues as well as future potential COVID-19 or other infectious disease challenges.

#### Data

Most participants reported the benefits of data to their work. Several participants remarked on the speed and frequency of data being produced during the pandemic, as a benefit. Several participants also reported the benefits of working with local data to manage outbreaks, a process which most participants agreed was best managed at a local level:

*“A lot of the information we got directly from the workplaces …So, we often didn’t have to wait for the national data to come through…actually we had good relationships with all our local schools, care homes, workplaces…especially with the schools and care homes we would normally know before the data came through nationally because people had already been in touch with us.”* (P1, PH)

Other participants also reported the benefit of regularly updated data from national organisations including the Health and Safety Executive (HSE) and Public Health England (PHE) (now UK Health Improvement Agency).

Several participants said that they felt that contact tracing was more effective when done at a local, or regional, level, rather than at a national level. They also reported that the implementation of a GM contact tracing hub had allowed them to engage with a larger proportion of residents. They suggested a number of reasons for this, including being able to draw on existing relationships with local communities in order to reach a greater number of residents, and being able to provide support for residents in order to help them to self-isolate if needed:

*“Once you actually had the resource at a GM or a local or even a neighbourhood level to actually go out and… use your knowledge of those communities …or the relationships that you’ve already got in place…that felt far more effective than…the national Serco call centre sort of trying to phone somebody up three times and if not getting through to them after the third time, or whatever, just considering it a dead lead...also I suppose then being able to almost have an intensive programme of support for somebody at a local or neighbourhood level... being able to provide that support or signpost to that support in a way that just wasn’t done at a national level. “ (*P15, local government)

#### Funding

Adequate funding was identified by several participants as a facilitator to managing COVID-19 transmission. Participants discussed accessing national level funds aimed at supporting local residents, including residents who were on on low incomes as well as those who were unable to work because they were self-isolating:

*“So, there was the Government Isolation Fund… that people could apply for, but we also had our local, and we called it a welfare fund, I think, that people could apply for as well.” (*P4, EH*)*

### Barriers to supporting employers to manage COVID -19 transmission

#### Issues around national guidance

Although several participants acknowledged that government advice had been helpful, many also reported barriers related to government guidance, which was often perceived as confusing or contradictory. This was exacerbated by the fact that the guidance was often released at very short notice, sometimes retrospectively. This gave participants very little time to read the guidance before they were expected to explain it to employers within their local authority area for immediate implementation. On other occasions, guidance was issued too late to be of assistance:

*“And we got no consultation obviously, which I understand because the government were having to react, but we literally found out about the legislation and guidance as the public did. So, immediately, we would get people…phoning, emailing, what does this mean for me, and can you explain this, you know, the whole argument. For us, it was very frustrating …. But obviously, we’re regulators and we had to.. enforce what we were being told to enforce.”* (P3, EH)

Other participants reported that the constant rule changes made it challenging to ensure that resources were accessible for a diverse population, including allocating time to translate guidance into multiple languages:

*“We were translating things into 21 different languages…And by the time you’ve done all that, the guidance or the rules have changed and you’re starting all over again.*” (P7, PH)

Numerous participants indicated that the government advice was ‘one size fits all’ and not always tailored to suit the diverse needs of all businesses. Other participants reported that guidance was being interpreted differently by different local authorities. This situation presented challenges for larger or national organisations who were liaising with multiple EH teams across the country:

*“Whether it's pub chains or national care home providers, they would be on the receiving end of different interpretations of the guidance, and they would just get increasingly frustrated with us.”* (P13, PH)

Some participants described the need on occasion to deviate from government advice, as shown in the quote below in relation to schools. Participants also reported surpassing national requirements at a GM level concerning care homes:

*“Particularly when we were having huge waves …it was like, oh, we’re stopping that…you don’t need to wear masks anymore, and we were having the biggest outbreaks in schools we’ve ever had and we were like, that decision isn’t supporting us. So, there were a couple of times when as DPH XX and other colleagues in GM overruled national and said actually for us, we want you to continue wearing face coverings in schools because we’ve got these huge outbreaks and we’re trying to keep you in school for your education. So, we did have to sort of make our own local decisions at times when it didn’t feel right for us, which ruffled some feathers at some times, but it was right for us.” (*P7, PH)

Several participants suggested that issues around compliance worsened through the changing stages of the pandemic and particularly towards the end of the pandemic. Participants reported that reinstating measures after they had been relaxed proved challenging and acted as a barrier to reducing transmission due to lower compliance levels:

*“When we were then starting to reintegrate after June, and we were starting to slightly increase some of the capacities within the …but then we moved into suddenly Plan B measures in the winter period and then we were having to sort of go back and reverse it. It was very difficult from a cultural point of view to then step back from where we were moving back to reintegration, to then step back into this sort of greater restrictions that we were putting into place for staffing. I think that to-ing and fro-ing really created quite a few difficulties for us in how we managed that.”* (P5, EH)

#### Access to data

Although access to data was described by participants as a facilitator to supporting workplaces, many participants also discussed difficulties in accessing data. Several participants reported that accessing data on areas with the highest rates of transmission, for example, proved particularly difficult in the early stages of the pandemic. Some participants voiced concerns that the available data lacked sufficient detail, whilst several others indicated that they were unable to obtain data in a timely manner. This delay in accessing data was often attributed to challenges surrounding data sharing agreements. Many participants also expressed frustrations related to contact tracing data nationally, especially at the outset of the pandemic. However, as discussed in the section on facilitators, issues associated with contact tracing had been alleviated to some extent by the implementation of a GM contact tracing hub. Several participants reported that with the cessation of testing, access to data became extremely limited:

*“I can’t not mention contact tracing…contact tracing sat with my environmental health officers, and it was a fiasco… all I was being told by my environmental health manager was …it doesn’t really work, we’re not getting the information when we need to get it.*” (P17, EH)

#### Insufficient funding

Although funding was recognised as a facilitator for supporting workplaces, some participants reported that measures such as access to COVID-19 grants had not been sufficient to offer adequate financial security for communities in deprived areas.

#### Ongoing COVID-19 challenges at the time of data collection

During the data collection period, for the majority of participants, the situation regarding the pandemic was described as ‘business as usual’ with no testing or tracing activities being conducted. Several participants suggested that future policies were focused and heavily reliant on vaccination as the primary strategy for preventing transmission. Some participants expressed concerns, however, that reliance on vaccines might lead to the neglect of other infection control measures. Others emphasised that differences in vaccination rates between groups (e.g. different occupational or age groups) would exacerbate health inequalities if vaccines were the sole COVID-19 mitigation measure. Several participants also reported a decrease in vaccination rates:

*“I’m not sure of the figures, but I suspect the uptake of the latest booster isn’t up there as in previous numbers as well. I speak to a lot of people and a lot of people are telling me they’re not having the booster. I think there’s a bit of resistance now in terms of the stories, oh I’ve had my booster and I was really, really ill, and all that sort of stuff. And this notion that well, I’ve had the booster, but I still got it, so what’s the point.” (*P16, EH)

Other participants suggested that future challenges related to COVID-19 included broader challenges around health inequalities and existing problems in the health structure. They also highlighted potential challenges in addressing the needs of people who were experiencing Long COVID. This is demonstrated in the quote below which relates to rehabilitation services for people with Long COVID:

*“The other thing that we haven’t invested in in this country, and we didn’t have enough of previously were therapy and rehab services. So, now we’ve got a whole load of more people needing rehab, but we don’t have anything like the services that we need.”* (P1, PH)

#### Non COVID-19 Challenges and priorities

Several participants indicated that managing the impacts of COVID had become less all-consuming than previously. They discussed balancing continuing COVID impact alongside managing non-COVID challenges and priorities. Non-COVID challenges reported at the time of data collection included the cost of living and energy crises, austerity, and the war in Ukraine. They also included the backlog in health service demand, dealing with other illnesses such as monkeypox, and addressing the impacts of COVID-19 lockdowns and other restrictions on the education system:

*“I think we’re back to our main challenges, from a PH perspective, which are things like housing and employment and poverty and cost of living and all those kind of things…infectious diseases-wise, actually we’re dealing with Scarlet Fever and invasive Group A Strep infections, and Monkey Pox and stuff like that. So, COVID just kind of sits alongside those other infectious diseases really, but probably more like, it’s more akin to how we manage flu now.”* (P2, PH)

Similarly, EH participants discussed supporting employers to comply with EH legislation that could not prioritised at the height of the pandemic:

*“Our focus now in my teams has been very much on trying to recover because, as I said at the start, our bread and butter for the environmental health team is inspection work of food businesses, and they were pretty much left to their own devices for 18 months. And we’re seeing a huge fallout from that. So, all of our kind of time and effort now is focused on trying to get around all of the food businesses and bring them back up to standard where needed. “* (P3, EH).

### Resilience: building on lessons learnt during the course of the pandemic

Participants discussed building on opportunities created and lessons learnt during the course of the pandemic. These included building on partnerships that were developed during the pandemic, as well as applying learning about controlling the spread of COVID-19 in order to tackle respiratory infections more broadly.

One of the most significant and positive changes reported by participants was new partnerships and ways of working developed as a result of dealing with COVID-19 that were continuing to be maintained for good practice in other areas:

*“I think what’s been good is that we built relationships with key people really, so we know that if we’ve got any future situations, not just COVID-19 but other things, that we’ve got those relationships in place, we can get in contact people, we can come together quickly, and we can just get on with whatever we’re faced. So, I think that’s a real benefit from the COVID work forced people to work together in a way, we had to come together, but we have built good partnerships and built good relationships that we can build on, continue to build on.” (*P7, PH)

*“So, we still have that relationship and we’ve expanded it beyond COVID, and now we’ve got this, kind of, like almost personal relationship where you see people, oh we haven’t met in person, but it was awesome the way that we really worked together during the pandemic.”* (P12, PH)

Other participants suggested that there was broader learning and future work around partnerships to prevent infection transmission:

*“I do think there’s a lot of positives. I do think there’s a lot of opportunities the pandemic has given us. I think it has heightened awareness of the spread of respiratory infections, the importance of environmental cleaning, the importance of just handwashing and the basics. I think there’s opportunities to almost use that to say remember how bad COVID was let's not get there, let’s wash our hands. It’s that sort of thing that you can use. I do think…I noticed on a practical level in lots of workplaces, they’ve kept quite a few of the practices they had. It might be that it does make sense, they noticed the drop in absences, so let's keep those workstations spaced out, let’s keep those Perspex screens in.“* (P10, PH)

However, several participants asserted that, in addition to the pandemic having an unequal impact on certain groups, not all workers would be able to benefit equally from the lessons learnt during the pandemic:

*“The way we work is much more flexible and movable then it was. And again that helps us to manage transmission of all infections... So I think that's a positive in terms of working cultures and practices. I suppose the risk in that is again the inequality, people with a nice office-based job like myself is very different to someone who’s got to be in work, who’s got to be in maybe quite tough conditions day in day out.. Might not get very good sick pay, if any at all. So there's still some of those same challenges there and the inequality that could result….I think there’s both challenges but opportunities as well going forward.”* (P10, PH)

Participants suggested that lessons learnt during the pandemic, including increased awareness of the barriers and facilitators to being able to support employers, can be used to address other health challenges and to prepare for future pandemics and health crises.

## Discussion

These findings build on and concur with the findings of previously published PROTECT research that examined perspectives of DsPH on the reasons for COVID-19 transmission [4]. The current study also adds the perspectives of local authority PH and EH teams, and provides insights based on local authority teams’ experiences of managing COVID-19 transmission in GM, an area that was disproportionately affected by the pandemic [8], both before and after publication of the government report ‘Living with COVID’ [14].

In the current study, both the qualitative and quantitative findings suggested that many workplaces had reverted to ‘business as usual’ at the time of data collection in the ‘Living with COVID’ phase of the pandemic. Interview participants discussed other challenges that local authorities were now managing alongside COVID-19, including the impact of the cost-of-living crisis, and challenges related to managing Long COVID. A number of barriers and facilitators to controlling transmission were identified by research participants, and interview participants discussed how the lessons learnt during the pandemic were being used to improve other health outcomes, and to prepare for future health crises.

Qualitative data explored a wide range of responses to reduce COVID-19 prevalence rates and prevent outbreaks. Within local authority roles this included helping employers to understand national guidance, as well as providing support with testing, contact tracing and vaccination. This concurs with the findings of previous PROTECT research that examined perspectives of DsPH on the reasons for COVID-19 transmission [4], as well as wider literature [10].

Both qualitative and quantitative data outlined the extent to which a range of measures introduced during the course of the pandemic had reduced following the 2022 ‘Living with COVID’ [14] report. Measures that had reduced included hygiene measures, physical and social distancing, and improvements to workplace ventilation.

The critical role of workplace ventilation in ventilation in reducing transmission risk has been highlighted in the literature [14] and was acknowledged in the interviews as becoming more widely recognised as an important mitigation measure. However, the surveys suggest ventilation measures implemented during the pandemic were not very widespread and had reduced greatly by the data collection period, with only 9.2% employees and 15.2% of employers reporting that their workplaces currently provided better ventilation at the time of data collection. However, it is possible that ventilation measures were in place that employees were unaware of.

There were some differences between sectors with regards to continuing mitigations, with interview participants reporting that mitigations were continuing in sectors such as the health sector, including in care homes, at the time of data collection in Autumn 2022. Recent research has suggested that long term mitigation measures in some workplaces may be warranted, especially in the education and care sectors, where research suggests that workers have persistently higher risks [2]. Several interview participants, however, suggested that the COVID-19 vaccine was the only mitigating measure that was still in place, with some suggesting that there was an ‘over reliance’ on the vaccine as a way of controlling transmission rates.

Levels of job stress appear to have remained higher than prior to the pandemic, with almost half of employees surveyed finding their job very or extremely stressful, at the time of data collection in 2022. Strategies to minimise stress and enhance resilience in the workforce should be a key consideration in managing future outbreaks [26].

Interview participants identified several facilitators for reducing COVID-19 prevalence. These included good communication, funding, and timely access to data, along with new and enhanced partnership working. Participants also emphasised the benefits of interventions such as contact tracing being delivered at a local level. This approach allows them to draw on existing relationships with local communities to reach a greater number of residents and to provide support for residents in order to help them to self-isolate if needed. This aligns with the findings of wider research [3, 27] and research conducted as part of PROTECT [4] which highlighted the importance of local approaches to provide tailored support for those being disproportionately affected by COVID-19.

Partnerships at the local, regional and national levels were key facilitators reported by interview participants who benefitted from internal and external partnerships including those with statutory, community and health organisations. PH participants discussed working closely with EH colleagues, as well as with a wide range of other stakeholders including local GM police and CCGs. Participants also worked closely with local communities, including with community and voluntary organisations and faith leaders. Participants also discussed partnership working with employers to reduce transmission as well as employers working together to discuss strategies to reduce transmission.

These findings align with previous literature [10] which suggests that partnerships are key to future success in managing COVID-19 transmission risk, as well as improving population health more generally. The *GM Strategy*, ‘Our People Our Place' continues to emphasise the importance of partnerships between civic leaders, businesses, and the wider community.

Participants also discussed a number of barriers to reducing prevalence, which included confusing or contradictory national guidance which changed frequently and at short notice. Other barriers included delayed access to data and issues with data sharing. Participants also highlighted inequalities and deprivation levels as a barrier, as local level interventions to reduce transmission rates were limited by structural, systematic inequalities which were difficult to resolve with local level resources in the short term. The literature (e.g. 4,12) suggests that socio-demographic factors impact the transmission of COVID-19, and this is supported by data from this research.

This findings of this study concur with the findings of previously published PROTECT research that examined perspectives of DsPH on the reasons for COVID-19 transmission [4]. Qualitative data from the current project, which was based on the experiences of PH and EH more broadly as well as DsPH, suggested that people who did not receive sick pay, who were on zero-hours contracts, had precarious employment terms, were self-employed but did not qualify for support grants, or were in low-income jobs, found it more difficult to take time off work in order to self-isolate, which may have also made them hesitant to take a COVID-19 test. The extent of this problem is suggested by the findings of the employee survey which showed that almost 6% of employees surveyed had gone into work despite being asked to isolate.

As reported in previous research [28], again including research conducted as part of PROTECT [4] participants also highlighted the intersection of other factors, including ethnicity and age, with factors such as employment on wider prevalence rates. For example, several interview participants said that people from certain ethnic minority groups might be more likely to be in frontline jobs where they were unable to work from home. Respondents suggested people working as taxi drivers as an example of this. In the surveys, however, there were too few respondents from ethnic minority backgrounds to explore this further.

Five point nine percent of respondents to the employee survey had attended work despite being asked to isolate, and this was more common among workers from lower-income groups. Along with qualitative data from interviewees, which emphasised the importance of adequate financial support for self-isolation, this demonstrates the need for sufficient funding in the case of managing future outbreaks and ensuring compliance.

The current study suggests that COVID-19 exacerbated existing inequalities in health. This aligns with wider research such as research conducted by Bambra et al. [29] who suggested that COVID-19 interacts with and exacerbates people’s existing social conditions and diseases. They suggest that historically, pandemics have been experienced unequally with higher rates of infection and mortality among those from the most disadvantaged communities.

Interview participants also reported differences in vaccination rates between different groups. They highlighted that residents working in certain occupations (e.g., manual workers) and particularly people from certain age groups (e.g., younger people) and certain minority ethnic groups working in these occupations, were less likely to be vaccinated. In the employee survey, employees with a higher level of qualification were more likely to have had at least two vaccinations plus one or more boosters. This aligns with the findings of previous research—the OpenSAFELY project [30], for example, identified differences in vaccination rates based on ethnicity and across rankings of deprivation. The authors of that study concluded that reasons behind the differences in vaccination coverage were complex. However, in our employer survey, those with a lower income were more likely to have had these vaccinations – this unexpected result requires deeper investigation. It is plausible that this finding reflects the positive outcomes of the extensive efforts that have been directed towards supporting specific communities with vaccines.

Interview participants reported that local authorities were increasingly focussing on other priorities alongside COVID-19, now that it was less all-consuming. These priorities included the cost-of-living crisis and war in Ukraine, as well as other illnesses such as monkey pox, as well as efforts to increase vaccination rates for other illnesses.

Future challenges reported by interview participants included responding to people who were experiencing Long COVID. The employee survey revealed that levels of Long COVID were high, with almost 1 in 4 (23%) of those who responded to the survey describing themselves as having the condition. The GM residents’ survey also revealed high levels of Long COVID, with around four in ten respondents (40%) who previously had COVID-19 reporting they are still experiencing impacts as a direct result of this [30]. National figures [31] also suggest that in November 2022, around 3.4% of the population as a whole were experiencing self-reported Long COVID.

As discussed earlier in the paper, the Government report ‘Living with COVID’ [11] suggests that the future path of the virus is currently uncertain, and therefore emphasises the importance of future planning in order to be prepared for possible resurgences of the virus or the emergence of potential future variants.

The research suggests that learning from the pandemic can be used to address other health outcomes and to prepare for future health crises. Interview participants highlighted, for example, that partnerships that they had formed during the pandemic could be focussed on these other health issues and were in place ready to address future pandemics. This aligns with the findings of wider research. Coleman et al. [32], for example, based on their research across eight work sectors, which was conducted as part of PROTECT, reported that most employers felt better prepared for future health emergencies. However, interview participants, in alignment with the findings of wider research, emphasised that it is important this is supported by adequate funding, including funding for local authority teams [4, 33].

### Limitations of the research

Seventy percent of respondents to the employee survey reported that they worked in education, which may have skewed the findings and meant that the survey was not as representative as it could have been. Most employee survey respondents had a degree or higher qualification, which excluded the views of a large segment of the working population. This is likely to be explained by variations in co-operation from different unions in recruiting respondents, with the teaching unions providing the most support. Another potential factor could be the data collection methods employed, as the survey was conducted online, which might have discouraged individuals without high qualifications from participating due to the assumed level of literacy required. Furthermore, very few of the employee survey participants were from minority ethnic backgrounds, and a larger proportion of the participants were women. Additionally, very few of the survey respondents were on precarious employment contracts.

The employee and employer surveys, along with the interviews, included questions about different time points throughout the pandemic. However, the surveys and interviews were only conducted on one occasion and therefore they provide a’snapshot’ of respondents’ experiences at the time of data collection in Autumn 2022. Responses also relied on participants’ recollection when reporting prior events. If the surveys and interviews had been conducted at different time points, responses might have varied.

## Recommendations

Based on the interviews with local authority teams and the two online surveys, the research team make a number of recommendations for national government and local authority teams. Recommendations include:

- ➢ Further funding for areas of development, including continued funding for PH and EH teams to enable them to continue working collaboratively in order to support employers
- ➢ Provision of adequate financial support to enable people to self-isolate when necessary
- ➢ Continued work to support specific risk sectors, including the food manufacturing sector as well as schools and care homes, in managing the transmission of respiratory illnesses including COVID-19
- ➢ Ongoing work to address health inequalities, including working with employers and national government to address factors influencing the transmission of infectious diseases like COVID-19, such as the absence of sick pay
- ➢ Building on and sharing examples of good practice to control transmission of COVID-19 within and across local authority areas
- ➢ Continued partnership working at local authority, regional and national levels, and with partners including health, police, local communities, and workplaces (large and small) to effectively manage transmission of COVID-19
- ➢ Continued development of inter-organisational strategies to manage COVID-19 transmission. These strategies should specifically incorporate measures to minimise stress and enhance resilience in the workplace to prepare for future outbreaks
- ➢ Encouragement (by local and national government) of enhanced workplace ventilation and other mitigation measures to prevent COVID-19 transmission
- ➢ Support for those who are experiencing Long COVID.

## Conclusion

The research reflects some of the key issues faced by the GM region in managing workplace transmission during the COVID-19 pandemic and on changes experienced and the future of ‘Living with COVID’.

Some key issues have arisen from this study which align with national research, as well as previous research conducted as part of the PROTECT project, such as the inequitable impact of the pandemic on those in lower socio demographic groups and people who are already experiencing health inequalities. The current research demonstrated the way this primarily impacted on workplace transmission through precarious working roles and different types of industries. The challenges identified by interview participants around controlling transmission in schools, care homes, food processing and call centres in particular draws attention to the need for careful planning for these areas. As a result, there is evidence of existing work continuing in these sectors to ensure ongoing commitment to infection prevention.

This work has highlighted the risks of poor communication or timeliness of guidance and a lack of joined up thinking or slow access to data. The study demonstrated the benefits of good quality local data facilitated by excellent local partnerships and a sense of community and collaborative working. Participants discussed partnerships that had been established during the pandemic and had now been broadened to address other health issues. Others discussed partnerships that were now in place ready to tackle future pandemics or other health crises. It is vital therefore to draw on lessons learned throughout the pandemic to address wider health issues and to ensure preparedness for any future events.

## Supplementary Information

Supplementary Material 1.

Supplementary Material 2.

Supplementary Material 3.

Supplementary Material 4.

Supplementary Material 5.

Supplementary Material 6.

## Data Availability

The datasets generated and/or analysed during the current study are not publicly available due in order to protect participant confidentiality - anonymising the files would require significant redaction, and consent was not sought from the participants to make qualitative data publicly available but are available from the corresponding author on reasonable request.

## Abbreviations

ADPH: Association of Directors of Public Health
CCG: Clinical Commissioning Group
DsPH: Directors of Public Health
EH: Environmental Health
GM: Greater Manchester
HSE: Health and Safety Executive
ONS: Office for National Statistics
PH: Public Health
PHE: Public Health England
PROTECT: Partnership for Research in Occupational, Transport and Environmental COVID-19 Transmission
SAGE: Scientific Advisory Group for Emergencies
