# Supplementary material for: An investigation of the impact of ‘Living with COVID’ on workplace COVID-19 transmission risk, response and resilience - lessons learnt and future challenges
Source: BMC Public Health. 2024 Oct 18;24:2871. doi: 10.1186/s12889-024-20320-3 (PMC11488279; doi:10.1186/s12889-024-20320-3)
Supplement: Supplementary file 1 — Supplementary Material 1. [file 12889_2024_20320_MOESM1_ESM.pdf]

GM Covid Public Health (Employers)

Start of Block: Default Question Block

Gm survey employer pis v1.2 25th july **Background Information & Consent**

You are being invited to take part in a survey which is part of a research study looking at the impact of the COVID-19 pandemic in Greater Manchester workplaces. The research aims to understand the impact of recent changes to government policy on perceptions of risk of transmission of COVID-19, wellbeing, and safety in the workplace. Your views are important as they will be used to inform future guidance on COVID-19 transmission mitigation measures. This survey is for people who are employers (owners or senior managers) in Greater Manchester workplaces.

Please take time to read the attached participant information sheet carefully before deciding whether to take part. By completing and returning the attached survey you give the following consent: 1. I confirm that I have read the participant information sheet Gm survey employer pis v1.2 25th july for this study and have had the opportunity to consider the information and ask questions and had these answered satisfactorily.
2. I understand that my participation in the study is voluntary and that I am free to withdraw at any time without giving a reason and without detriment to myself.
3. I understand that it will not be possible to remove my data from the project once it has been anonymised and forms part of the data set.
4. I agree that any data collected may be included in an anonymised form in reports, journal publications or conference presentations.
5. I understand that data collected during the study may be looked at by individuals from The University of Manchester or regulatory authorities, where it is relevant to taking part in this research. I give my permission for these individuals to have access to my data.
6. I agree that any anonymised data collected may be made available to other researchers and may be uploaded to a data repository
7. I agree that any data collected will be archived and may be used as anonymous data as part of a secondary data analysis process.
Data Protection The personal information we collect and use to conduct this research will be processed in accordance with data protection law as explained in the Participant Information Sheet and the Privacy Notice for Research Participants

○ Click here if you agree to take part (1)

End of Block: Default Question Block

Start of Block: Section 1 - About you and your organisation

Q1 1. Please confirm where in the Greater Manchester Region your organisation is located. (We are only asking employers located in this area to complete the questionnaire)

- Bolton (2)
- Bury (3)
- Oldham (4)
- Rochdale (5)
- Stockport (6)
- Tameside (7)
- Trafford (8)
- Wigan (9)
- Salford (10)
- City of Manchester (11)
- Unsure (but within Greater Manchester) (12)
- Not located within Greater Manchester (13)

Skip To: End of Survey If 1. Please confirm where in the Greater Manchester Region your organisation is located. (We are on... = Not located within Greater Manchester

Q2 2. Which of the following sectors does your organisation work in?

○ A - Agriculture, forestry and fishing (1)

○ B - Mining and quarrying (2)

○ C - Manufacturing (3)

○ D - Electricity, gas, steam and air conditioning supply (4)

○ E - Water supply; sewerage, waste management and remediation activities (5)

○ F - Construction (6)

○ G - Wholesale and retail trade; repair of motor vehicles and motorcycles (7)

○ H - Transportation and storage (8)

○ I - Accommodation and food service activities (9)

○ J - Information and communication (10)

○ K - Financial and insurance activities (11)

○ L - Real estate activities (12)

○ M - Professional, scientific and technical activities (13)

○ N - Administrative and support service activities (14)

○ O - Public administration and defence; compulsory social security (15)

○ P - Education (16)

○ Q - Human health and social work activities (17)

○ R - Arts, entertainment and recreation (18)

○ S - Other service activities (19)

○ T - Employed by a household as domestic staff (inlcuding producing goods/services for a household for their own use) (20)

○ U - International organisations and bodies - includes for example the United Nations, the World Bank and the European Free Trade Association as well as diplomatic activities and consular missions) (21)

○ Unsure (22)

Q3 3. How many employees are there in the organisation you work for?

○ 0-9 (1)

○ 10-49 (2)

○ 50-249 (3)

○ 250+ (4)

○ Don't know / unsure (5)

Q4 4. How many people are employed at the site where you usually work?

○ 0-9 (4)

○ 10-49 (5)

○ 50-249 (6)

○ 250+ (7)

○ Don't know / unsure (8)

Q5 5. How many sub-contractors work for the organisation?

- 0-9 (4)
- 10-49 (5)
- 50-249 (6)
- 250+ (7)
- Don't know / unsure (8)

Q6 6. How many sites does the organisation have?

- 0 (1)
- 1 (2)
- 2 (3)
- 3-5 (4)
- 6-10 (5)
- 11-20 (6)
- 20-100 (7)
- 100+ (8)

Q7 7. What is your role/job title?

____

Page Break

Q8 8. Please give a brief overview of types of job roles of people who work at the company (beyond standard professional services such as HR, Admin, Finance)

____

Q9 9. What is your employment status/tenure?

- ◯ Employed full time (Permanent) (1)
- ◯ Employed full time (Temporary) (2)
- ◯ Employed part-time (Permanent) (3)
- ◯ Employed part-time (Temporary) (4)
- ◯ Self-employed (5)
- ◯ Sub-contractor (6)

Q10 10. Do you live in Greater Manchester?

- ◯ Yes (7)
- ◯ No (8)

Page Break

End of Block: Section 1 - About you and your organisation

Start of Block: Section 2 - About your organisation policies

Q11 11. What was the company policy on working from home, including the proportion of time that employees are permitted to work from home? *(prior to the start of the pandemic)*

____

Q12 12. What was the company policy on working from home, including the proportion of time that employees are permitted to work from home? *(during the height of the pandemic in 2020/21?)*

____

Page Break

Q13 13. What is the company policy on working from home, including the proportion of time that employees are permitted to work from home? *(currently in 2022?)*

____

Q14 14. *Prior to the pandemic*, what proportion of their usual pay did employees receive when they were absent from work due to sickness? (e.g. SSP, full pay etc.)

____

Q15 15. *During the height of the pandemic in 2020/21*, what proportion of their usual pay did employees receive when they were absent from work due to sickness? (e.g. SSP, full pay etc.)

____

Q16 16. *Currently in 2022*, what proportion of their usual pay do employees receive when they were absent from work due to sickness? (e.g. SSP, full pay etc.)

____

Q17 17. *During the height of the pandemic in 2020/21*, what proportion of their usual pay did employees receive if they had to isolate as a close contact of someone with COVID-19? (e.g. SSP, full pay etc.)

____

Q18 18. *Currently in 2022*, what proportion of their usual pay do employees receive if they have to isolate as a close contact of someone with COVID-19? (e.g. SSP, full pay etc.)

____

Q19 19. *During the height of the pandemic in 2020/21*, did the company provide accommodation for employees?

____

Q20 20. *Currently in 2022*, does the company provide accommodation for employees?

____

Q21 21. *During the height of the pandemic in 2020/21*, did the company provide any employees with transport to work?

____

Q22 22. *Currently in 2022*, does the company provide any employees with transport to work?

____

End of Block: Section 2 - About your organisation policies

Start of Block: Section 3 - About your workplace (and related environmental factors)

Q23 23. Do any of the following apply in your workplace? (select all that apply)

	Prior to the pandemic (1)	At the height of the pandemic in 2020/21 (2)	Currently in 2022 (3)
Shift working (1)	○	○	○
Cold environment (2)	○	○	○
Employees come into close contact with colleagues ((3)	○	○	○
Employees have face to face contact with members of the public as part of their role (4)	○	○	○
Employees travel as part of the business day (e.g between sites) (5)	○	○	○

End of Block: Section 3 - About your workplace (and related environmental factors)

Start of Block: Section 4 - About transmission risk control measures

Q25 25. Please select which of the following measures your employer implemented at your workplace to reduce the risk of COVID-19 infection.

	During the pandemic in 2020/21 (1)	Currently in 2022 (2)
Reducing contact with surfaces that could be contaminated (1)	☐	☐
Reducing physical contact with colleagues (2)	☐	☐
Encouraging social distancing with colleagues (3)	☐	☐
Reducing physical contact with members of the public (4)	☐	☐
Enhanced hand washing facilities (5)	☐	☐
Provision of hand sanitisers (6)	☐	☐
Improving workplace cleaning (7)	☐	☐
Formation of work team bubbles (8)	☐	☐
Reduction of number of workers for specific tasks (9)	☐	☐
Screens or physical barriers (10)	☐	☐
Reducing the number of people at your workplace (11)	☐	☐
Enabling working from home (12)	☐	☐
Staggered start and finish times (13)	☐	☐

Workplace training on managing COVID-19 transmission (14)	☐	☐
Access restrictions to canteens, site, changing facilities (15)	☐	☐
Encouraging staff to wear face masks or other protective equipment (16)	☐	☐
Regular workplace testing for COVID-19 (17)	☐	☐
COVID-19 testing at home before attending the workplace (18)	☐	☐
Workplace COVID-19 Vaccination (19)	☐	☐
Providing better workplace ventilation (20)	☐	☐
Any other measures (please state) (21)	☐	☐
No measures implemented (22)	☐	☐

Q26 26. What, if any, training have employees received about reducing COVID-19 transmission in the workplace?

____

Q27 27. How well do you feel that employees in general are/were able to adhere to these measures?

- ○ 0 - Not at all (9)
- ○ 1 (10)
- ○ 2 (11)
- ○ 3 (12)
- ○ 4 (13)
- ○ 5 - Very well (14)

Q28 28. Were there any barriers to employees adhering to safety measures in your workplace during the pandemic in 2021/22?

- ○ Yes (1)
- ○ No (2)

Skip To: End of Block If 28. Were there any barriers to employees adhering to safety measures in your workplace during the... = No

---

Q28a 28a) What types of barriers were there to employees adhering to safety measures in the workplace during the pandemic? Please give examples if possible

____

End of Block: Section 4 - About transmission risk control measures

---

Start of Block: Section 5 - About your perception of transmission risk

Q29 29. Have there been any previous COVID-19 outbreaks in this workplace?

◯ Yes (1)

◯ No (2)

◯ Unsure (3)

Skip To: Q30 If 29. Have there been any previous COVID-19 outbreaks in this workplace? = No

Skip To: Q30 If 29. Have there been any previous COVID-19 outbreaks in this workplace? = Unsure

Q29a 29a. How many outbreaks of COVID-19 did you have in your workplace?

____

Q29b 29b. For each outbreak, how many employees were involved and which internal and external partners and organisations did you liaise with to control the outbreak?

____

Q30 30. How worried were/are you about COVID-19 transmission in the workplace?

	Not at all concerned 1 (1)	2 (2)	3 (3)	4 (4)	Very concerned 5 (5)
At the height of the pandemic in 2020/21 (1)	◯	◯	◯	◯	◯
Currently in 2022 (2)	◯	◯	◯	◯	◯

End of Block: Section 5 - About your perception of transmission risk

Start of Block: You've reached the end. Thanks for taking the time to complete the survey.

The end

Prize draw

If you would like to enter a prize draw to thank you for participating please follow this link: Prize Draw

Resource List Please find below a list of resources if you are feeing distressed, unwell, or concerned.
You are welcome to print or save this list.

If you are feeling unwell and fell concerned that you may have Corona virus (COVID-19), phone NHS 111 for advice or access NHS website
NHS website (http://www.nhs.uk/Pages/HomePage.aspx)
You can also get information about conditions, symptoms, treatments, and medicines For information about COVID-19 (e.g., symptoms, testing, self-isolation, people at higher risk), please go to the NHS website:
https://www.nhs.uk/conditions/coronavirus-COVID-19/
For information about current COVID-19 guidelines, please go to the Government website:
https://www.gov.uk/coronavirus

If you have concerns about your physical or mental health, please contact your GP: Call your GP surgery Visit your GP surgery's website (https://www.nhs.uk/service-search/find-a-gp) Use an online service to contact your GP (https://www.nhs.uk/using-the-nhs/nhs-services/gps/gp-online-services/) If you want alternative sources of support, please consider these services: Bereavement services (https://www.gov.uk/find-bereavement-services-from-council) Drug and alcohol services (Turning Point; https://www.turning-point.co.uk) Relationship support (Relate; https://www.relate.org.uk) Mental health support (MIND; 0300 123 3393; http://www.mind.org.uk/)

○ Please click here to end the survey (1)

End of Block: You've reached the end. Thanks for taking the time to complete the survey.
