# Supplementary material for: An investigation of the impact of ‘Living with COVID’ on workplace COVID-19 transmission risk, response and resilience - lessons learnt and future challenges
Source: BMC Public Health. 2024 Oct 18;24:2871. doi: 10.1186/s12889-024-20320-3 (PMC11488279; doi:10.1186/s12889-024-20320-3)
Supplement: Supplementary file 2 — Supplementary Material 2. [file 12889_2024_20320_MOESM2_ESM.pdf]

GM Covid Public Health Survey (Employees)

Start of Block: Default Question Block

*

Background Information & Consent

You are being invited to take part in a survey which is part of a research study looking at the impact of the COVID-19 pandemic in the workplace. The research aims to understand the impact of recent changes to government policy on perceptions of risk of transmission of COVID-19, wellbeing, and safety in the workplace. Your views are important as they will be used to inform future guidance on COVID-19 transmission mitigation measures. Please take time to read the attached participant information sheet carefully before deciding whether to take part.
By completing and returning the attached survey you give the following consent:
1. I confirm that I have read the participant information sheet Gm survey employee pis 1.2 11th july for this study and have had the opportunity to consider the information and ask questions and had these answered satisfactorily.
2. I understand that my participation in the study is voluntary and that I am free to withdraw at any time without giving a reason and without detriment to myself.
3. I understand that it will not be possible to remove my data from the project once it has been anonymised and forms part of the data set.
4. I agree that any data collected may be included in an anonymised form in reports, journal publications or conference presentations.
5. I understand that data collected during the study may be looked at by individuals from The University of Manchester or regulatory authorities, where it is relevant to taking part in this research. I give my permission for these individuals to have access to my data.
6. I agree that any anonymised data collected may be made available to other researchers and may be uploaded to a data repository
7. I agree that any data collected will be archived and may be used as anonymous data as part of a secondary data analysis process.
Data Protection The personal information we collect and use to conduct this research will be processed in accordance with data protection law as explained in the Participant Information Sheet and the Privacy Notice for Research Participants.

○ Click here if you agree to take part (1)

End of Block: Default Question Block

Start of Block: Section 1 - About you

Q1 1. Please confirm that you worked in the Greater Manchester Region at any point since April 2020. (If you did not we do not need you to complete the questionnaire)

○ Yes (1)

○ No (2)

Skip To: End of Survey If 1. Please confirm that you worked in the Greater Manchester Region at any point since April 2020.... = No

Q2 2. Do you live in the Greater Manchester area?

○ Yes (1)

○ No (2)

Q3 3. Which of the following sectors do you work in?

- ○ A - Agriculture, forestry and fishing (1)
- ○ B - Mining and quarrying (2)
- ○ C - Manufacturing (3)
- ○ D - Electricity, gas, steam and air conditioning supply (4)
- ○ E - Water supply; sewerage, waste management and remediation activities (5)
- ○ F - Construction (6)
- ○ G - Wholesale and retail trade; repair of motor vehicles and motorcycles (7)
- ○ H - Transportation and storage (8)
- ○ I - Accommodation and food service activities (9)
- ○ J - Information and communication (10)
- ○ K - Financial and insurance activities (11)
- ○ L - Real estate activities (12)
- ○ M - Professional, scientific and technical activities (13)
- ○ N - Administrative and support service activities (14)
- ○ O - Public administration and defence; compulsory social security (15)
- ○ P - Education (16)
- ○ Q - Human health and social work activities (17)
- ○ R - Arts, entertainment and recreation (18)
- ○ S - Other service activities (19)
- ○ T - Activities of households as employers; undifferentiated goods- and services-producing activities of households for own use (20)

○ U - Activities of extraterritorial organizations and bodies (21)

○ Unsure (22)

Q4 4. What size is the organisation you work for?

○ Micro (0-9 employees) (1)

○ Small (10-49 employees) (2)

○ Medium (50-249 employees) (3)

○ Large (250+ employees) (4)

○ Don't know / unsure (5)

Q5 5. How many people work at the site where you are based?

____

Q6 6. What is your job title?

____

Q7 7. What is your employment status?

- Employed (full-time) permanent contract (1)
- Employed (full-time) temporary (2)
- Employed (part-time) permanent contract (3)
- Employed (part-time) temporary (4)
- Self-employed (5)
- Sub-contractor (6)

Q8 8. How many hours are you contracted to work each week?

____

Q9 9. Do you supervise or manage other members of staff?

- Yes (1)
- No (2)

Skip To: Q10 If 9. Do you supervise or manage other members of staff? = No

---

Q9a 9a How many other members of staff do you manage or supervise?

- 0 (7)
- 1-5 (1)
- 6-10 (2)
- 11-50 (3)
- 51-100 (4)
- 101-500 (5)
- 500+ (6)

Q10 10. Do you have other forms of employment?

- Yes (1)
- No (2)

Q11 11. How long have you worked for this employer?

____

Q12 12. What is your annual income?

- ○ less than £15,000 (1)
- ○ £15,000 - £19,999 (2)
- ○ £20,000 - £29,999 (3)
- ○ £30,000 - £39,999 (4)
- ○ £40,000 - £49,999 (5)
- ○ £50,000 - £59,999 (6)
- ○ £60,000 - £69,999 (7)
- ○ £70,000 - £99,999 (8)
- ○ £100,000 - £149,999 (9)
- ○ £150,000+ (10)

Q13 13. What is your highest level of qualification?

- ○ Level 4 or above (Degree) (1)
- ○ Level 3 (A level or equivalent) (2)
- ○ Level 2 (GCSE or equivalent) (3)
- ○ Below level 2 (less than 5 GCSEs A-C) (4)
- ○ No qualifications (5)
- ○ Other (6) ____

Q14 14. How old are you?

- ○ 18 - 24 (1)
- ○ 25 - 34 (2)
- ○ 35 - 44 (3)
- ○ 45 - 54 (4)
- ○ 55 - 64 (5)
- ○ 65+ (6)

Q15 15. What is your gender?

- ○ Male (1)
- ○ Female (2)
- ○ Non-binary / third gender (3)
- ○ Prefer not to say (4)

Q16 16. What is your ethnicity?

- ○ 1. White - English / Welsh / Scottish / Northern Irish / British (1)
- ○ 2. White - Irish (2)
- ○ 3. White - Gypsy or Irish Traveller (3)
- ○ 4. Any other White background, please describe (4) ________________
- ○ 5. Mixed / Multiple - White and Black Caribbean (5)
- ○ 6. Mixed / Multiple - White and Black African (6)
- ○ 7. Mixed / Multiple - White and Asian (7)
- ○ 8. Any other Mixed / Multiple ethnic background, please describe (8) ________________
- ○ 9. Asian / Asian British - Indian (9)
- ○ 10. Asian / Asian British - Pakistani (10)
- ○ 11. Asian / Asian British - Bangladeshi (11)
- ○ 12. Asian / Asian British - Chinese (12)
- ○ 13. Any other Asian background, please describe (13) ________________
- ○ 14. Black / Black British - African (14)
- ○ 15. Black / Black British - Caribbean (15)
- ○ 16. Any other Black / African / Caribbean background, please describe (16) ________________
- ○ 17. Arab (17)
- ○ 18. Any other ethnic group, please describe (18) ________________

End of Block: Section 1 - About you

Start of Block: Section 2 - About your experience of COVID-19

Q17 17. During the pandemic, were you contacted by letter or text message to say you are at severe risk from COVID-19 due to an underlying health condition and should be shielding?

- Yes (1)
- No (2)

Q18 18. Is there anyone in your immediate family / 'bubble' who is more than usually vulnerable to COVID-19 (e.g., clinically vulnerable; has an underlying health condition)

- Yes (1)
- No (2)

Q19 19. Do you know or think that you have had COVID-19?

- Yes (confirmed with positive test) (1)
- Yes (suspected but not confirmed with test) (2)
- No (3)

Skip To: Q20 If 19. Do you know or think that you have had COVID-19? = No

Q19a. 19a. Which month/year did you have COVID-19?

____

Q20 20. Would you describe yourself as having ‘long COVID’ – that is, you are still experiencing symptoms more than 4 weeks after you first had COVID-19, that are not explained by something else?

- ○ Yes (confirmed by medical professional) (1)
- ○ Yes (suspected but not confirmed by a medical professional) (2)
- ○ No (3)

Q21 21. Have you been vaccinated against the virus that causes COVID-19?

- ○ Yes, I’ve had one dose (1)
- ○ Yes, I’ve had two doses (2)
- ○ Yes, I’ve had two doses and a booster (3)
- ○ Yes, I have had two doses and two boosters (9)
- ○ No, but I will get vaccinated once I am able to do so (5)
- ○ No, I have refused the vaccine (6)
- ○ Prefer not to say (7)
- ○ Other (8) ____

Q22 22. During the course of the pandemic, did you attend your workplace despite being asked to self-isolate?

- ○ Yes (1)
- ○ No (2)

Skip To: Q24 If 22. During the course of the pandemic, did you attend your workplace despite being asked to self-... = No

---

Q23 23 How many times did you attend your workplace despite being asked to self-isolate?

____

Q24 Q24 How many people including yourself live in your house with you?

- ○ At the height of the pandemic (1) ____
- ○ Currently (2) ____

Q25 25. Do you/have you claimed any of the following benefits from DWP?

	Prior to the pandemic (1)	During the pandemic in 2020/21 (2)	Currently in 2022 (3)
No benefits (1)	☐	☐	☐
Universal Credit. (2)	☐	☐	☐
Income-based Jobseekers Allowance. (3)	☐	☐	☐
Income-related Employment and Support Allowance. (4)	☐	☐	☐
Income Support. (5)	☐	☐	☐
Working Tax Credit. (6)	☐	☐	☐
Child Tax Credit. (7)	☐	☐	☐
Pension Credit. (8)	☐	☐	☐

End of Block: Section 2 - About your experience of COVID-19

Start of Block: Section 3 - About your workplace (and related environmental factors)

Q26 26. What proportion of your working time is spent indoors?

	0 10 20 30 40 50 60 70 80 90 100
Out of 100% ()	

Q27 27. Were you able to work from home prior to the start of the pandemic?

- ◯ Yes (1)
- ◯ No (2)

Q28 28. If you work mainly (more than 50%) indoors, what would best describe your place of work (choose only one)

- ◯ Personal Office (1)
- ◯ Shared office (2)
- ◯ Open plan office (3)
- ◯ Warehouse (4)
- ◯ Working from home (5)
- ◯ Other (please specify) (6) ____

Q29 29. How many people usually share the space you work in, including co-workers and clients?

- ◯ Work alone (1)
- ◯ 1-2 people (2)
- ◯ 3-5 people (3)
- ◯ 6-20 people (4)
- ◯ 20-50 people (5)
- ◯ 50-100 people (6)
- ◯ 100+ (7)

Q30 30. Does your main job include working in direct contact with members of the public?

- Yes (1)
- No (2)

Q31 31. Does your main job require working in direct contact with people with COVID-19? (for example as a health care worker or carer)

- Yes (1)
- No (2)

Q32 32. Do you live with anyone you work with?

- Yes (workplace accommodation) (1)
- Yes (accommodation not managed by the workplace) (2)
- No (3)

Q33 33. How do you usually travel to/from work?

- ◯ Private car/van (alone) (1)
- ◯ Private car/van (travel with others) (2)
- ◯ Work car/van (alone) (3)
- ◯ Work car/van (travel with others) (4)
- ◯ Public transport (5)
- ◯ N/A as working from home (6)
- ◯ Other (please specify) (7)

Q34 34. Do you have access to sick pay?

- ◯ Yes (1)
- ◯ No (2)

Skip To: Q35 If 34. Do you have access to sick pay? = No

Q34a 34a. What type of sick pay would you be able to claim?

- ◯ Statutory Sick Pay (SSP) (1)
- ◯ Company - Basic (2)
- ◯ Company - Enhanced (3)
- ◯ Unsure (4)

Q35 35. Did/do you get sick pay if you are isolating?

	During the height of the pandemic in 2020/21 (1)	Now in 2022 (2)
Yes (1)	○	○
No (2)	○	○
Don't know (4)	○	○

End of Block: Section 3 - About your workplace (and related environmental factors)

Start of Block: Section 4 - About transmission risk control measures

Q36 36. Please select which of the following measures your employer implemented at your workplace to reduce the risk of COVID-19 infection.

	During the pandemic in 2020/21 (1)	Currently in 2022 (2)
Reducing contact with surfaces that could be contaminated (1)	☐	☐
Reducing physical contact with colleagues (2)	☐	☐
Encouraging social distancing with colleagues (3)	☐	☐
Reducing physical contact with members of the public (4)	☐	☐
Enhanced hand washing facilities (5)	☐	☐
Provision of hand sanitisers (6)	☐	☐
Improving workplace cleaning (7)	☐	☐
Formation of work team bubbles (8)	☐	☐
Reduction of number of workers for specific tasks (9)	☐	☐
Screens or physical barriers (10)	☐	☐
Reducing the number of people at your workplace (11)	☐	☐
Enabling working from home (12)	☐	☐
Staggered start and finish times (13)	☐	☐
Workplace training on managing COVID-19 transmission (14)	☐	☐

Access restrictions to canteens, site, changing facilities (15)	☐	☐
Encouraging staff to wear face masks or other protective equipment (16)	☐	☐
Regular workplace testing for COVID-19 (17)	☐	☐
COVID-19 testing at home before attending the workplace (18)	☐	☐
Workplace COVID-19 Vaccination (19)	☐	☐
Providing better workplace ventilation (20)	☐	☐
Any other measures (please state) (21)	☐	☐
No measures implemented (22)	☐	☐

Q37 37. Do **you** adhere to the control measures at work?

- ○ Always (1)
- ○ Most of the time (2)
- ○ About half the time (3)
- ○ Sometimes (4)
- ○ Never (5)

Q38 38. Do **others** adhere to the control measures at work?

- ○ Always (1)
- ○ Most of the time (2)
- ○ About half the time (3)
- ○ Sometimes (4)
- ○ Never (5)

Q39 39. As far as you're aware, has your employer conducted a COVID-19 risk assessment of your workplace? (This would include, for example, identifying activities/situations that might cause transmission, how likely it is someone could be exposed, and identifying actions that could reduce the risk)

- ○ Yes (1)
- ○ No (2)
- ○ Don't know (3)

Skip To: End of Block If 39. As far as you're aware, has your employer conducted a COVID-19 risk assessment of your workpl... = No

Skip To: End of Block If 39. As far as you're aware, has your employer conducted a COVID-19 risk assessment of your workpl... = Don't know

---

Q40a 39a. Did you contribute to this COVID-19 risk assessment, either personally or via a third party?

- ○ Yes (1)
- ○ No (2)

End of Block: Section 4 - About transmission risk control measures

---

Start of Block: Section 5 - About your perception of transmission risk

Q40 40. How concerned are you currently in 2022 about…

	Not at all concerned 1 (1)	2 (2)	3 (3)	4 (4)	Very concerned 5 (5)
a) yourself contracting COVID-19 (1)	○	○	○	○	○
b) a family member contracting COVID-19 (2)	○	○	○	○	○
c) COVID-19 occurring in your workplace (3)	○	○	○	○	○
d) COVID-19 remaining as a health issue (4)	○	○	○	○	○

Q42 41. How concerned were you in 2020/21 about…

	Not at all concerned 1 (1)	2 (2)	3 (3)	4 (4)	Very concerned 5 (5)
a) yourself contracting COVID-19 (1)	○	○	○	○	○
b) a family member contracting COVID-19 (2)	○	○	○	○	○
c) COVID-19 occurring in your workplace (3)	○	○	○	○	○
d) COVID-19 remaining as a health issue (4)	○	○	○	○	○

Q43 42. How likely do you think it is for you to get infected with COVID-19 currently in 2022?

	Not likely at all 1 (1)	2 (2)	3 (3)	4 (4)	Very likely 5 (5)	N/A (6)
a) Generally (1)	○	○	○	○	○	○
b) At work (2)	○	○	○	○	○	○
c) Compared to other persons (3)	○	○	○	○	○	○
d) At home (4)	○	○	○	○	○	○
e) On your commute to/from work (5)	○	○	○	○	○	○
f) On public transport (6)	○	○	○	○	○	○
g) Shopping (7)	○	○	○	○	○	○
h) At social events (8)	○	○	○	○	○	○
i) Indoors compared to outdoors (9)	○	○	○	○	○	○
j) Outdoor public gatherings (10)	○	○	○	○	○	○
k) Indoor public gatherings (11)	○	○	○	○	○	○
l) Within the home (12)	○	○	○	○	○	○

Q44 43. How likely do you think it was for you to get infected with COVID-19 at the height of the pandemic in 2020-21?

	Not likely at all 1 (1)	2 (2)	3 (3)	4 (4)	Very likely 5 (5)	N/A (6)
a) Generally (1)	○	○	○	○	○	○
b) At work (2)	○	○	○	○	○	○
c) Compared to other persons (3)	○	○	○	○	○	○
d) At home (4)	○	○	○	○	○	○
e) On your commute to/from work (5)	○	○	○	○	○	○
f) On public transport (6)	○	○	○	○	○	○
g) Shopping (7)	○	○	○	○	○	○
h) At social events (8)	○	○	○	○	○	○
i) Indoors compared to outdoors (9)	○	○	○	○	○	○
j) Outdoor public gatherings (10)	○	○	○	○	○	○
k) Indoor public gatherings (11)	○	○	○	○	○	○
l) Within the home (12)	○	○	○	○	○	○

Q45 44. Do you wear a face covering outside work?

- ○ Always (2)
- ○ Often (3)
- ○ Sometimes (4)
- ○ Rarely (5)
- ○ Never (6)

Q45a 44a. When do you wear a face covering outside work and why?

____

Q46 45. Do you agree or disagree with the following statements?
Currently...

	Strongly disagree 1 (1)	2 (2)	3 (3)	4 (4)	Strongly agree 5 (5)	Don't know (6)
Thinking of COVID-19, my supervisor/manager places a strong emphasis on workplace health and safety (1)	○	○	○	○	○	○
Safety regarding COVID-19 is given a high priority by my supervisor/manager (2)	○	○	○	○	○	○
My supervisor/manager considers safety relating to COVID-19 to be important (3)	○	○	○	○	○	○
Safety procedures and practices are sufficient to prevent COVID-19 outbreaks occurring. (4)	○	○	○	○	○	○
There is frequent communication about COVID-19 transmission in my workplace. (5)	○	○	○	○	○	○
Workers are regularly consulted about COVID-19 management. (6)	○	○	○	○	○	○
Workers have sufficient access to training on preventing transmission of COVID-19. (7)	○	○	○	○	○	○

All the people who work in my team are fully committed to preventing transmission of COVID-19. (8)	○	○	○	○	○	○
I use all the necessary COVID-19 safety equipment to do my job (9)	○	○	○	○	○	○
I use the correct safety procedures related to COVID-19 for carrying out my job (10)	○	○	○	○	○	○
I ensure the highest levels of COVID-19 safety when I carry out my job (11)	○	○	○	○	○	○
I can take time off work to self-isolate if needed. (12)	○	○	○	○	○	○
My manager/supervisor discusses COVID-19 health-related topics with me. (13)	○	○	○	○	○	○
My manager/supervisor routinely discusses with me which objectives are to be accomplished concerning worksite COVID-19 health promotion. (14)	○	○	○	○	○	○
My manager/supervisor invites me to contribute my experience towards the implementation of health promotion projects relating to COVID-19. (15)	○	○	○	○	○	○

My manager/supervisor reflects on how to increase COVID-19 health and safety at our department. (16)	○	○	○	○	○	○
We are informed about COVID-19 health issues in work meetings (17)	○	○	○	○	○	○
My manager/supervisor includes me in decisions concerning COVID-19 health issues. (18)	○	○	○	○	○	○
My manager/supervisor assumes responsibility for my health regarding COVID-19. (19)	○	○	○	○	○	○

End of Block: Section 5 - About your perception of transmission risk

---

Start of Block: Section 6 - About your wellbeing

Q47 46. Below are some statements about general wellbeing (overall not just at work). Please indicate what best describes your experience of each over the last two weeks.

	None of the time 1 (1)	2 (2)	3 (3)	4 (4)	All the time 5 (5)
I have been feeling optimistic about the future (1)	○	○	○	○	○
I have been feeling useful (2)	○	○	○	○	○
I have been feeling relaxed (3)	○	○	○	○	○
I have been dealing with problems well (4)	○	○	○	○	○
I have been thinking clearly (5)	○	○	○	○	○
I have been feeling close to other people (emotionally) (6)	○	○	○	○	○
I have been able to make up my own mind about things (7)	○	○	○	○	○

Q48 47. How stressful did you find your main job…

	Not at all stressful 1 (1)	Mildly stressful 2 (2)	Moderately stressful 3 (3)	Very stressful 4 (4)	Extremely stressful 5 (5)
Prior to the start of the pandemic (1)	○	○	○	○	○
During the height of the pandemic in 2020/21 (2)	○	○	○	○	○
Currently in 2022 (3)	○	○	○	○	○

End of Block: Section 6 - About your wellbeing

Start of Block: You've reached the end. Thanks for taking the time to complete the survey.

The end **Resource List**
Please find below a list of resources if you are feeing distressed, unwell, or concerned.
You are welcome to print or save this list.

If you are feeling unwell and fell concerned that you may have Corona virus (COVID-19), phone NHS 111 for advice or access NHS website
NHS website (http://www.nhs.uk/Pages/HomePage.aspx)
You can also get information about conditions, symptoms, treatments, and medicines For information about COVID-19 (e.g., symptoms, testing, self-isolation, people at higher risk), please go to the NHS website:
https://www.nhs.uk/conditions/coronavirus-COVID-19/
For information about current COVID-19 guidelines, please go to the Government website:
https://www.gov.uk/coronavirus

If you have concerns about your physical or mental health, please contact your GP: Call your GP surgery Visit your GP surgery's website (https://www.nhs.uk/service-search/find-a-gp) Use an online service to contact your GP (https://www.nhs.uk/using-the-nhs/nhs-services/gps/gp-online-services/) If you want alternative sources of support, please consider these services: Bereavement services (https://www.gov.uk/find-bereavement-services-from-council) Drug and alcohol services (Turning Point; https://www.turning-point.co.uk) Relationship support (Relate; https://www.relate.org.uk) Mental health support (MIND; 0300 123 3393; http://www.mind.org.uk/)

If you would like to enter the prize draw to win a shopping voucher please follow this link: Prize draw

○ Please click here to end the survey (1)

End of Block: You've reached the end. Thanks for taking the time to complete the survey.
