# Supplementary material for: An investigation of the impact of ‘Living with COVID’ on workplace COVID-19 transmission risk, response and resilience - lessons learnt and future challenges
Source: BMC Public Health. 2024 Oct 18;24:2871. doi: 10.1186/s12889-024-20320-3 (PMC11488279; doi:10.1186/s12889-024-20320-3)
Supplement: Supplementary file 3 — Supplementary Material 3. [file 12889_2024_20320_MOESM3_ESM.pdf]

Additional File 3 – Local authority participant roles

Participant code	Local authority team
P1, PH	Public Health
P2, PH	Public Health
P3, EH	Environmental Health
P4, EH	Environmental Health
P5, EH	Environmental Health
P6, PH	Public Health
P7, PH	Public Health
P8, EH	Environmental Health
P9, PH	Public Health
P10, PH	Public Health
P11, PH	Public Health
P12, PH	Public Health
P13, PH	Public Health
P14, EH	Environmental Health
P15, LG	Local Government
P16, EH	Environmental Health
P17, EH	Environmental Health
P18, EH	Environmental Health
P19, PH	Public Health
