# Supplementary material for: An investigation of the impact of ‘Living with COVID’ on workplace COVID-19 transmission risk, response and resilience - lessons learnt and future challenges
Source: BMC Public Health. 2024 Oct 18;24:2871. doi: 10.1186/s12889-024-20320-3 (PMC11488279; doi:10.1186/s12889-024-20320-3)
Supplement: Supplementary file 4 — Supplementary Material 4. [file 12889_2024_20320_MOESM4_ESM.docx]

### Additional File 4 - Interview schedule – local authority participants

1. What has your role been in terms of workplace transmission throughout the pandemic? How has this changed throughout the course of the pandemic?
2. How would you describe your local authority in terms of its population/level of deprivation/work sector profile etc.? How does this compare to neighbouring local authorities?
3. What, in your opinion, are the main factors that contribute to differences in the workplace transmission and outbreaks of COVID-19?

Prompts:

- In a previous PROTECT project, Directors of Public Health who we interviewed identified lack of access to sick pay, working on zero hours contracts or in insecure employment, making it more difficult for people to self-isolate when needed, as key risk factors related to employment. Structural factors including deprivation, employment, and housing, converging with demographic factors including ethnicity and age, and vaccination rates, influenced the wider picture of prevalence rates of COVID-19. What is your opinion on this statement?
- Do you think that the importance of any of the factors described above has changed during the course of the pandemic? For example, population factors, deprivation, nature of work, effectiveness of contact tracing, regional commutes between LAs?
- What is the interplay between the different factors?

1. How would you describe the changes in COVID-19 prevalence and outbreaks in your local authority over the course of the pandemic?
2. How have you identified particular workplaces for tailored interventions/support around reducing transmission and controlling outbreaks?

Prompts:

-Is this based on previous data about inequalities or new covid-related data?

-How have you dealt with challenges associated with reaching certain population groups (e.g., agency workers, sub-contractors) with your interventions?

-Have you noticed any variation in number of outbreaks according to size and type of workplace, or between wards/ geographical areas within your local authority?

1. How effective do you think that national level strategies, policies and guidance have been in preventing workplace transmission, and workplace outbreaks, during the course of the pandemic and currently?

Prompts:

-What have the most and least effective strategies/ policies: regulation, guidelines, fiscal measures, environmental/social planning, service provision, legislation, and communication/marketing been during the course of the pandemic?

-Has the effectiveness of strategies changed throughout the timeline of the pandemic?

-What has helped/hindered the introduction of these strategies (including resource implications)

1. How effective do you think that local level strategies, policies and guidance have been in preventing workplace transmission, and workplace outbreaks, during the course of the pandemic and currently?

Prompts:

- What have been the most and least effective local / regional strategies/ policies: regulation, guidelines, fiscal measures, environmental/social planning, service provision, legislation, and communication/marketing during the course of the pandemic?

- Has the effectiveness of strategies changed throughout the timeline of the pandemic?

- What has helped/hindered the introduction of these strategies?

-Is the effectiveness of strategies affected by specific characteristics of your localities, places, population, economies?

-What could be done to intervene earlier and curtail prevalence in regions?

1. Which (if any) organisations in Greater Manchester did you work with in terms of management of COVID-19 workplace transmission and outbreaks during the course of the pandemic, and currently in 2022?

Prompts:

-Which organisations have taken a lead? How have local organisations been working together? E.g., joint forums, with LAs / CCGs / ICSs, resilience hubs?

-Has there been any misalignment/conflict between local, regional, and national strategies/policies/guidance and how have you managed this in your LA?

**-**Similar industries grouped together / shared knowledge?

**-** Have there been opportunities for you to learn from other local authorities?

1. What data, evidence and knowledge is used to inform local decision making?

Prompts:

- What sources of data/information do you find useful?

-How is existing knowledge / data used to inform guidance?

- What are key data or knowledge gaps that need to be addressed?

-How could data be used to anticipate areas at greater risk from COVID prevalence in the future?

-Have there been any issues around data sharing (e.g., between partners)?

-Has behavioural science informed your approach to encouraging hand hygiene, physical distancing, wearing of face coverings, self-isolation, etc? If so, how?

1. Have there been any evaluation activities, or anything used to assess mitigations/strategy/policy either in acceptability within workplaces or effectiveness of COVID-19 workplace strategies?
2. In your opinion, what are the future challenges for preventing or reducing COVID-19 transmission in the workplace?

Prompts:

- How has what we have learned in the past going to benefit us in the future? planning for Winter 2022, emergence of new variants of COVID-19, support for people to self-isolate (e.g., financial support),impact of vaccination programme, national strategy for COVID-19 transmission management, resources, sustainability, anything else?

1. Would you be able to provide a brief overview of other challenges that local authority staff example) are facing?

Prompts:

1. How does management of COVID-19 rank alongside, and interact with, these other challenges? For example, the cost of living crisis, other illnesses such as monkeypox, cardiovascular disease.
2. What future research do you think would be most useful to provide insights that can support future practice and decision making?

Prompts:

- Access to data improves outcomes - do you agree?

- What are the key questions for research relating to controlling workplace outbreaks of COVID-19 (e.g., patterns/correlations that cannot be explained)?

1. Is there anyone else that you think we should speak to as part of this research?
2. Is there anything else that you think we need to know, or anything else that you would like to add?
