# Supplementary material for: An investigation of the impact of ‘Living with COVID’ on workplace COVID-19 transmission risk, response and resilience - lessons learnt and future challenges
Source: BMC Public Health. 2024 Oct 18;24:2871. doi: 10.1186/s12889-024-20320-3 (PMC11488279; doi:10.1186/s12889-024-20320-3)
Supplement: Supplementary file 5 — Supplementary Material 5. [file 12889_2024_20320_MOESM5_ESM.pdf]

PROTECT National Core Studies Project

Participant Information Sheet – Interviews with key stakeholders

Greater Manchester Case Study

About the research

You are being invited to take part in a survey which is part of a research study looking at the impact of the COVID-19 pandemic in the workplace. The research aims to understand the impact of recent changes to government policy on perceptions of risk of transmission of COVID-19, wellbeing, and safety in the workplace. Your views are important as they will be used to inform future guidance on COVID-19 transmission mitigation measures.

Before you decide whether to take part, it is important for you to understand why the research is being conducted and what it will involve. Please take time to read the following information carefully before deciding whether to take part and discuss it with others if you wish. Please ask if there is anything that is not clear or if you would like more information. Thank you for taking the time to read this.

What is the purpose of the research?

We aim to understand how COVID-19 has impacted organisations and employees work and perception of transmission risk in Greater Manchester. We also aim to identify effective control measures which contribute to COVD-19 safe workplace and which support employees' wellbeing and safety.

You have been invited because you have been identified as an employee at an organisation working in Greater Manchester and we are interested in your experience of your organisation's COVID-19 transmission management at work.

Who will conduct the research?

The research will be conducted by a team led by Prof Martie Van tongeren at the University of Manchester in collaboration with the Health and Safety Executive (HSE).

Who is funding the research project?

The research is funded by the Health and Safety Executive.

Will the outcomes of the research be published?

We will use data (in pseudo-anonymised form - each interview participant will be assigned a unique identifier) to inform our findings which will be written up in reports (to funders and the public), scientific publications and discussed in presentations and workshops.

Who has reviewed the research project?

The project has been reviewed by The University of Manchester Proportionate Research Ethics Committee.

What would I be asked to do if I took part?

We would like to invite you to participate in a conversational interview with one of our research team lasting approximately 1 hour duration (maximum). Interviews will be conducted remotely, either over the phone or via Zoom/Skype/Teams at a time suitable to yourself. The researcher will contact you before the agreed time to provide instructions for connecting for the interview / discussion. The consent form will be sent to you in advance of the interview. You will then have the option of adding your scanned signature to the consent form and returning it to us via email, or if you prefer we can take verbal consent at the start of the interview. The interview will be audio recorded with your consent.

Will I be compensated for taking part?

There will be no payment for your participation.

What happens if I do not want to take part or if I change my mind?

It is up to you to decide whether or not to take part. If you do decide to take part you will be asked to print off or save a copy of this given this information sheet. If you decide to take part you are still free to withdraw at any time without giving a reason and without detriment to yourself. However, it will not be possible to remove your data from the project after the draft project report has been written This does not affect your data protection rights. If you decide not to take part you do not need to do anything further. Your organisation will not be informed if you choose to participate or not.

Data Protection and Confidentiality

What happens to the data collected?

Audio recordings will only be accessible to the research team (University of Manchester). Some platforms (e.g. Zoom) record both video and audio at the same time, but we will only keep a sound file (any video file will be immediately deleted). If you are uncomfortable about this you will be able to turn off your video. Data from all interviews and discussion groups (once transcribed; the audio is copied out into a Word or similar document) and documentations (for context e.g. policies) collected will be analysed together. This will take place at the University of Manchester / researcher’s home (due to Covid restrictions) and will be conducted by the research team. All the data will be stored on the University of Manchester’s secure servers.

Will my participation in the study be confidential and my personal identifiable information be protected?

In accordance with data protection law, The University of Manchester is the Data Controller for this project. This means that we are responsible for making sure your personal information is kept secure, confidential, and used only in the way you have been told it will be used. All researchers are trained with this in mind, and your data will be looked after in the following way:

In order to undertake the research project and provide you with feedback (results) we will need to collect the following personal information/data about you:

- Name
- Job title
- Organisation
- Contact details (email, phone number, and/or postal address)

The anonymous dataset will be stored on secure University servers and retained for up to 10 years as per publisher guidance when pursuing the publication of study findings.

Please also note that individuals from The University of Manchester or regulatory authorities may need to look at the data collected for this study to make sure the project is being carried out as planned. This may involve looking at identifiable data. All individuals involved in auditing and monitoring the study will have a strict duty of confidentiality to you as a research participant.

When you agree to take part in a research study **anonymised** data may be shared with **researchers/researchers at other institutions**. We may also upload anonymised data to a Research Data Repository for transparency and to support further related research. Your information will only be used in accordance with The University of Manchester's Privacy Notice for Research**.** This information will not identify you and will not be combined with other information in a way that could identify you.

What are my rights in relation to the information you will collect about me?

You have a number of rights under data protection law regarding your personal information. For example, you can request a copy of the information we hold about you.

If you would like to know more about your different rights or the way we use your personal information to ensure we follow the law, please consult our Privacy Notice for Research.

What if I have a complaint?

Contact details for complaints

Minor complaints:

If you have a complaint that you wish to direct to members of the research team, please contact: **Prof Sheena Johnson Sheena.Johnson@manchester.ac.uk**

Formal complaints:

If you wish to make a formal complaint to someone independent of the research team or if you are not satisfied with the response you have gained from the researchers in the first instance then please contact

The Research Ethics Manager, Research Office, Christie Building, The University of Manchester, Oxford Road, Manchester, M13 9PL, by emailing: research.complaints@manchester.ac.uk or by telephoning 0161 306 8089.

If you wish to contact us about your data protection rights, please email dataprotection@manchester.ac.uk or write to The Information Governance Office, Christie Building, The University of Manchester, Oxford Road, M13 9PL at the University and we will guide you through the process of exercising your rights.

You also have a right to complain to the Information Commissioner's Office about complaints relating to your personal identifiable information Tel 0303 123 1113

Contact Details

If you have any queries about the study or if you are interested in taking part then please contact the researcher(s)

Dr Claire Mann Claire.Mann@manchester.ac.uk

Cath Lewis cath.lewis@manchester.ac.uk

Janet Ubido janet.ubido@manchester.ac.uk
