# Supplementary material for: An investigation of the impact of ‘Living with COVID’ on workplace COVID-19 transmission risk, response and resilience - lessons learnt and future challenges
Source: BMC Public Health. 2024 Oct 18;24:2871. doi: 10.1186/s12889-024-20320-3 (PMC11488279; doi:10.1186/s12889-024-20320-3)
Supplement: Supplementary file 6 — Supplementary Material 6. [file 12889_2024_20320_MOESM6_ESM.docx]

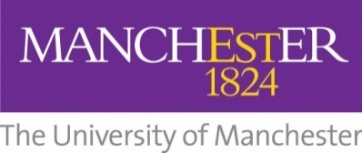


**Greater Manchester Case Study**

**CONSENT FORM (Interviews with key stakeholders)**

**If possible, please scan in your signature and return this form to the research team before your interview. If this is not possible, we can take consent verbally at the start of the interview**.

If we take consent verbally (having read the participant information sheet sent to you and having had a chance to ask any questions) this will be recorded separately to the questions we ask to preserve confidentiality.

**Please initial box**

|  | **Activities** | Initials |
| --- | --- | --- |
| 1 | I confirm that I have read the attached information sheet (Version xx. Date: XX/XX/XXXX**)** for the above study and have had the opportunity to consider the information and ask questions and had these answered satisfactorily. |  |
| 2 | I understand that my participation in the study is voluntary and that I am free to withdraw at any time without giving a reason and without detriment to myself. I understand that it will not be possible to remove my data from the project once it has been anonymised and forms part of the data set.  I agree to take part on this basis. |  |
| 3 | I agree to the interview being audio recorded. |  |
| 4 | I agree that any data collected may be published in anonymous form in academic books, reports or journals. It will not be possible to identify me personally from any study material. |  |
| 5 | I understand that data collected during the study may be looked at by individuals from The University of Manchester or regulatory authorities, where it is relevant to my taking part in this research. I give permission for these individuals to have access to my data. |  |
| 6 | I agree that non-personal information, i.e. information collected in the interview, can be shared with the other researchers (as detailed in the Participant Information Sheet). |  |
| 7 | I agree that the researchers may retain my contact details in order to provide me with a summary of the findings for this study. |  |
| 8 | I agree that any data collected will be archived and may be used as anonymous data as part of a secondary data analysis process. |  |
| 9 | I agree to take part in this study. |  |

**The personal information we collect and use to conduct this research will be processed in accordance with data protection law as explained in the Participant Information Sheet and the** [**Privacy Notice for Research Participants**](http://documents.manchester.ac.uk/display.aspx?DocID=37095)**.**

|  |  |  |  |  |
| --- | --- | --- | --- | --- |
| Name of participant |  | Date |  | Signature |
| Name of researcher |  | Date |  | Signature |
